# Association Between Type of Infertility and Live Birth in Couples With a Single Intrauterine Insemination Resulting in Pregnancy: A Propensity Score Matching Cohort Study

**DOI:** 10.3389/fendo.2022.926183

**Published:** 2022-07-14

**Authors:** Wen He, Song Chen, Jianping Huang, Xiaofang Zhang, Lili Hu, Zhigang Xue, Yu Qiu

**Affiliations:** ^1^ Department of Regenerative Medicine, Tongji University School of Medicine, Shanghai, China; ^2^ Department of Orthopaedic Trauma, East Hospital, Tongji University School of Medicine, Shanghai, China; ^3^ Reproductive Medicine Center, Ji’an Women and Child Health care Hospital, Jiangxi, China; ^4^ Reproductive Medicine Center, Tongji Hospital, Tongji University School of Medicine, Shanghai, China

**Keywords:** primary infertility, secondary infertility, ovarian stimulation, intrauterine insemination, live birth

## Abstract

**Background:**

Few studies have described the relationship between the type of infertility and live birth in patients treated with intrauterine insemination (IUI). We focused on this issue and attempted to explore it.

**Methods:**

This retrospective study enrolled 2,256 infertile patients who underwent their first IUI cycle and were subsequently diagnosed with a clinical pregnancy at Ji’an Women and Child Health Care Hospital between 2007 and 2018. Inductees were divided into primary infertility (1,680 patients) and secondary infertility groups (876 patients). Following 1:1 propensity score matching to obtain balanced data, the COX proportional hazards model, landmark analysis, and subgroup analysis were used to assess the association between infertility types and live birth rates. Subsequently, a sensitivity analysis was employed to evaluate the potential effect of unmeasured confounding on outcomes.

**Results:**

Of the 1,486 patients who were identified as a matched cohort, 743 were in the primary infertility group and the remaining patients were in another group. A total of 1,143 patients had live births during 431,009 person-days of follow-up (average 290.0 days). Throughout the follow-up period, patients with secondary infertility demonstrated more live births than patients with primary infertility (hazard ratio [HR], 1.16; 95% confidence interval [CI], 1.04 to 1.30; *P* = .007). More details were observed in the landmark analysis. Live birth rates were similar in both groups within 316 days of follow-up (HR, 0.84; 95% CI, 0.62 to 1.14; *P* = .269), whereas the opposite was found between 316 days of follow-up and delivery day (HR, 1.19; 95% CI, 1.06 to 1.34; *P = .*004). This was also obtained in a subgroup analysis of patients younger than 35 years old and patients treated with natural cycles (NCs) and IUIs.

**Conclusion:**

Among the infertile patients who underwent a single natural or stimulated cycle followed by IUI and had later pregnancies, full-term young secondary infertility mothers (<35 years of age) had a greater chance of having viable babies than the primary infertility ones. The latter may get more benefits when undergoing ovarian stimulation and IUI rather than NC-IUI.

## Introduction

Infertility is defined as the inability to conceive within one year of regular unprotected intercourse and can be classified as primary or secondary infertility depending on whether the couple has a previous history of a successful pregnancy (1). It has been recognized as a worldwide public health issue for over a decade by the World Health Organization (WHO) (2). Annually, more than 70 million couples worldwide may suffer social exclusion, divorce, financial, mental, or other health consequences, such as unfavorable pregnancy outcomes and subsequent adult illnesses, due to their inability to have children (3–6). Clearly, if infertility is well intervened, these dilemmas will disappear. Otherwise, the situation may become more critical as the number of infertility cases increases.

At present, therapeutic options for infertile couples include assisted reproductive technologies, such as *in vitro* fertilization (IVF) and embryo transfer, as well as intrauterine insemination (IUI). In most countries, the high cost and limited insurance coverage of IVF make it an unattainable option for most infertile couples, indirectly making IUI the first-line treatment option for managing infertility (7, 8). In addition, IUI is more flexible and can be performed with or without ovarian stimulation (OS), making it simple to repeat the procedure even if the woman does not conceive.

Although IUI has been a commonly used treatment for many years and has been widely reported, the effect of the type of infertility on pregnancy outcome remains controversial (9–11). Furthermore, most of these studies have focused on the relationship between infertility types and pregnancy rates. However, to our knowledge, few studies have been interested in the relationship between the type of infertility and live birth. This article, therefore, performed to examine the differences in live births between patients with primary and secondary infertility, with a view to contributing to this field in a modest way. Based on the findings of Dinelli et al. (9), namely that pregnancy rates after IUI are higher in patients with secondary infertility than in patients with primary infertility, we proposed and tested the hypothesis that live birth rates might also be higher in women with secondary infertility than in those with primary infertility.

## Materials and Methods

### Data Sources and Study Population

This study is a retrospective cohort analysis using information obtained from the electronic medical record database of Ji’an Women and Child Health Care Hospital, a level one infertility center in Jiangxi, China. The database contains patient data that include population characteristics at hospital admission, reproductive history, infertility assessment results, treatment procedures, and relevant clinical outcomes. Related dates are also available. The study was approved by the institutional review board of Ji’an Women and Child Health Care Hospital, with a waiver of informed consent because all data were deidentified. This research followed the Strengthening the Reporting of Observational Studies in Epidemiology (STROBE) reporting guideline for cohort studies (12).

To minimize the likelihood of confounding in this study, only infertile couples who underwent a single either natural or stimulated cycle followed by intrauterine insemination (IUI) and possessed clinical pregnancy were enrolled from July 2007 to November 2018. Women were between 18 and 40 years of age, had a normal uterine cavity with at least one patent fallopian tube confirmed by hysterosalpingograms (HSG), and had a male partner with a semen specimen of at least 10 million sperm per milliliter. Patients were excluded if they did not meet the inclusion criteria or had crucial data absent. None of the couples using donor semen had any involvement in this trial. Finally, a total of 2,781 couples who experienced treatments with clinical pregnancy were identified. Of those, 225 patients (8.1% of all eligible) were excluded owing to missing data on baseline demographic characteristics or cycles lost to follow-up, leaving 2,556 persons for the current analyses.

### Treatment Protocols

Prior to treatment, the couple underwent an infertility evaluation including a HSG and at least two semen analyses. Body mass index (BMI) was determined for each patient as well. On day 3 of the menstrual cycle, serum estradiol and progesterone levels were obtained. Additionally, baseline transvaginal ultrasounds were performed to assess antral follicle count (AFC). The total number of follicles measuring 2 to 9 mm on both ovaries were tallied to constitute the basal AFC.

Subsequently, infertility diagnoses were determined by the patient’s physician based on reproductive history and results of the infertility assessment, which included diminished ovarian reserve, endometriosis, hypothalamic amenorrhea, and other ovulatory dysfunctions, male factor infertility, sexual dysfunction (male and/or female), unexplained infertility, and uterine and/or cervical factor, and were categorized into four specific groups in this study as following: maternal factor, paternal factor, combined and unexplained infertility. Then, individualized treatment protocols for IUI cycles were made as mutual agreements between couples and their primary physicians. Patients with regular menstrual cycles (28–35 days) were recommended for natural cycles (NC). Others were assigned to perform three types of ovarian stimulation (OS) options with oral clomiphene citrate (CC) (Fertilan, Cyprus), injectable gonadotropins (Gn) (Livzon, Zhuhai, China), or both. The initial dose was 50 mg/day for CC (days 3–8) or of 75 IU/day for Gn, and the dosage was modulated by the woman’s age, indication, rank of the attempt, and previous responses to stimulation. The initial dose of Gn was maintained for the first 6 days of stimulation, after which it was adapted according to the ovarian response.

In patients with NC, monitoring of follicular growth was initiated in the mid-follicular phase and performed every 1-3 days, depending on the dominant follicle size. Ovulation trigger of recombinant human chorionic gonadotropin (hCG) was administered when at least one dominant follicle ≥18 mm in diameter appeared. Endometrial thickness was also measured at this time point. Then, Insemination was performed approximately 36 hours later or 24 hours after a spontaneous LH rise (≥35 IU/L). Otherwise, IUI cycles were cancelled according to the criteria that the number of mature follicles was 0 or more than 3 (13). If the follicle grew on the side of the obstructed fallopian tube, the circle was also cancelled. As for patients treated by OS plans, monitoring was performed starting on cycle days 11 to 12 until a dominant follicle (≥18 mm) was identified. Ovulation trigger and IUI were implemented as described in the NC protocol.

### Semen Preparation and Intrauterine Insemination

After 3 to 7 days of abstinence and 2 hours prior to insemination, semen samples were collected at the laboratory and then kept liquefaction at 37°C for 30 minutes. The semen was washed with 2-layer density gradient centrifugation and followed by a count and motility evaluation (14, 15). Before the start of IUI procedure, patient and partner identification were confirmed. Each operation to gently place a dose of prepared sperm (0.5mL) into the lower uterine segment of a patient was completed by one of the center’s gynecologists in an artificial insemination room with a soft catheter (Cook Group, USA). At the end of the procedure, patients were advised to lay supine for half an hour. Luteal support was routinely offered to all patients with oral dydrogesterone (Duphaston, Abbott Biologicals, USA) 10 mg twice daily from the day after IUI for 14 days.

### Pregnancy Diagnosis and Follow Up

A serum beta hCG pregnancy test would be conducted two weeks after insemination. The presence of a positive result (>10 IU/L) was considered as a biochemical pregnancy. Once the pregnancy was confirmed chemically, an ultrasound would be scheduled two weeks later to determine location of the pregnancy and the number of implantation sites. Findings of at least one intrauterine pregnancy sac could be described as clinical pregnancy.

Prenatal records were requested from the treating obstetrician. Each prenatal visit was kept in the electronic medical record database after the prenatal encounter, until delivery. Moreover, delivery outcome records were obtained from the labor & delivery department where the patient gives birth to her baby(s).

### Exposure Variable and Outcomes

The exposure was the type of infertility in this study. Definitions as previously stated. Eligible patients were selected into either the primary infertility group or the secondary infertility group. Delivery outcomes for the first IUI cycle leading to clinical pregnancy were compared between the two groups. The outcome was all live births in women with different infertility types. Patients with clinical miscarriage, ectopic pregnancy, or stillbirth in this trial were categorized as censored to perform a time-to-event analysis. Live birth was defined as the delivery of a live-born infant after 28 weeks of gestation or more. The absence of signs of life at or after birth was considered as still birth. Clinical miscarriage described pregnancies in which an intrauterine gestational sac was identified on ultrasound but the pregnancy did not progress or was spontaneously lost. Ectopic pregnancy is one in which the blastocyst implants at any site other than the endometrial lining of the uterus cavity.

### Statistical Analysis

Patient’s characteristics collected at baseline were the following: maternal age, smoking status, BMI, infertility duration, infertility causes, treatment protocol, year of treatment, postwash total motile sperm count (PTMSC), baseline serum estradiol, progesterone levels and transvaginal ultrasound findings.

All baseline characteristics were transformed into categorical variables and data were presented as numbers and percentages. Differences in baseline characteristics between the two groups were compared with the chi-square or Fisher exact test as appropriate. Furthermore, due to the nonrandom assignment of patients, propensity score matching was performed to balance the potential differences and minimize selection biases associated with retrospective data analysis. Propensity score was estimated using logistic regression analysis based on the patient’s baseline characteristics mentioned above. One-to-one nearest-neighbor matching method was used to match patients with a predefined caliper width equal to 0.02 of the standard deviation (SD) of the logit of the propensity score (16). No replacement was allowed, and patients were matched only once. For matched covariates, a standardized difference of less than 10% indicates a relatively small imbalance (17). The paired McNemar test was also conducted to evaluate balance in the matched cohort.

For time-to-event analysis, the endpoint in this study was defined as the date of delivery for patients. The length of observation from the day of beginning treatment to the arrival of the endpoint was described as the main time variable. The main event was the presence of a live birth, and other events such as miscarriage, ectopic pregnancy, or stillbirth were considered as right censoring. The Kaplan–Meier curves with log-rank test were employed to report live births in matched sets of infertility types. Cumulative incidence was plotted as 1 minus Kaplan-Meier estimates. When two curves cross, this may indicate that the proportional hazards (PH) assumption is not met and a two-stage test would be an alternative (18, 19). Subsequently, univariable and multivariable Cox proportional hazards regression models with robust sandwich estimates were assessed to determine the factors that were associated with live birth (20). The PH assumption was examined using the Schoenfeld residuals test and log minus log plots (21, 22). If the condition is not met, landmark analysis will be taken (23). Moreover, subgroup analysis for live birth and were stratified by maternal age and treatment protocols. The consistency of hazard ratios (HRs) across subgroups was tested by the significance of the interaction terms, and the subgroup analyses were regarded as exploratory. Finally, a formal sensitivity analysis, as described by Tyler J. VanderWeele and Peng Ding (24), to assess the potential effect of unmeasured confounding on our results.

As to ensure the parsimony of the multivariable models described above, the baseline variables for inclusion were carefully chosen, that is to say, only variables that were considered clinically relevant or that had a P value less than.10 on the univariate analysis were entered into the final model. All tests were paired and two-sided, with a significance threshold of *P* <.05. The propensity score matching, two-stage test, Fine and Gray test, landmark analysis, and sensitivity analysis were performed using the statistical computing program R (version 4.0.2; http://www.r-project.org) with packages shown in the **Supplemental Table S1**, and the remaining analyses were performed using Stata software (version 14.0; Stata Corp., LLC) and SPSS software (version 22.0; IBM Corp., USA).

## Results

### Study Population

In total, 2,556 patients received their first IUI cycle and were subsequently diagnosed with a clinical pregnancy with complete medical records. Of these, 182 patients (8.1%) were aged 35 years or older, 809 patients (31.7%) had a history of infertility more than 3 years, 721 patients (28.2%) experienced unexplained infertility, and 690 patients (27.0%) were provided the treatment protocol of NC. Among all patients, 1,680 were allocated to the primary infertility group and the rest to the secondary infertility group. In this unmatched cohort, variables that differed between the two groups included maternal age, infertility duration, infertility causes, antral follicle count (AFC), endometrial thickness, PTMSC and year of treatment. Patients in the primary infertility group compared with those in the secondary infertility group were more likely to have AFC <11 (497 patients [29.6%] vs 165 patients [18.8%]) or >17 (316 patients [18.8%] vs 267 patients [30.5%]) on day 3 of menstrual cycle, PTMSC ≤38 (505 patients [30.1%] vs 177 patients [20.2%]) or ≥70 million (505 patients [30.1%] vs 177 patients [20.2%]), and endometrial thickness <8 mm(335 patients [19.9%] vs 285 patients [32.5%]) measured with a transvaginal ultrasound scan on the day of ovulation trigger. Then, through a 1:1 propensity score matching, 743 patient pairs were produced. Furthermore, standardized differences in baseline characteristics did not exceed 10% and no significant differences were observed between matched groups, implying that the covariates were well balanced (**Table 1**; **Supplementary Figure S1** and **Figure S2**).

**Table 1:** Baseline demographic characteristics of patients before and after propensity score matching based on infertility types.

Characteristic	Before propensity score matching			After propensity score matching					
	No. (%)			No. (%)					
	Primary infertility (n = 1680)	SecondaryInfertility (n = 876)	P value*	Primary infertility (n = 743)		SecondaryInfertility (n = 743)		P value*	
Maternal age range, yrs			<0.001					0.805	
<30	996 (59.3)	420 (47.9)		374 (50.3)		390 (52.5)			
30-34	584 (34.8)	374 (42.7)		312 (42.0)		301 (40.5)			
≥35	100 (6.0)	82 (9.4)		57 (7.7)		52 (7.0)			
Smoking	37 (2.2)	21 (2.4)	0.754	17 (2.30)		19 (2.6)		0.868	
BMI, kg/m^2^			0.604					0.887	
≤18.4	159 (9.5)	74 (8.5)		68 (9.15)		63 (8.5)			
18.5-23.9	668 (39.8)	344 (39.3)		300 (40.4)		296 (39.8)			
24.0-27.9	540 (32.1)	302 (34.5)		243 (32.7)		251 (33.8)			
≥28	313 (18.6)	156 (17.8)		132 (17.8)		133 (17.9)			
Infertility duration, yrs			<0.001					0.708	
≤3	1080 (64.3)	667 (76.1)		547 (73.6)		540 (72.7)			
>3	600 (35.7)	209 (23.9)		196 (26.4)		203 (27.3)			
Infertility causes			<0.001					0.898	
Maternal factor	671 (39.9)	447 (51.0)		358 (48.2)		366 (49.3)			
Paternal factor	167 (9.9)	127 (14.5)		95 (12.8)		98 (13.2)			
Combined	312 (18.6)	111 (12.7)		102 (13.7)		101 (13.6)			
Unexplained	530 (31.5)	191 (21.8)		188 (25.3)		178 (24.0)			
FSH/LH ratio†			0.232					1.000	
<3	1487 (88.5)	789 (90.1)		672 (90.4)		671 (90.3)			
≥3	193 (11.5)	87 (9.9)		71 (9.6)		72 (9.7)			
Estradiol, pg/ml†			0.702					0.643	
<30	328 (19.5)	178 (20.3)		149 (20.1)		148 (19.9)			
30-60	929 (55.3))	490 (55.9)		399 (53.7)		419 (56.4)			
>60	423 (25.2)	208 (23.7)		195 (26.2)		176 (23.7)			
AFC†			<0.001					0.651	
<11	497 (29.6)	165 (18.8)		165 (22.2)		154 (20.7)			
11-17	867 (51.6)	444 (50.7)		379 (51.0)		394 (53.0)			
>17	316 (18.8)	267 (30.5)		199 (26.8)		195 (26.2)			
Endometrial thickness, mm§			<0.001					0.577	
<8	335 (19.9)	285 (32.5)				200 (26.9)			
≥8	1345 (80.1)	591 (67.5)				543 (73.1)			
DFC‡			0.522					0.659	
1	1103 (65.7)	564 (64.4)		477 (64.2)		486 (65.4)			
≥2	577 (34.3)	312 (35.6)		266 (35.8)		257 (34.6)			
PTMSC, millions			<0.001					0.478	
≤38	505 (30.1)	177 (20.2)		175 (23.6)		167 (22.5)			
38.1-58.9	396 (23.6)	218 (24.9)		182 (24.5)		185 (24.9)			
52.0-69.9	412 (24.5)	245 (28.0)		194 (26.1)		199 (26.8)			
≥70.0	367 (21.8)	236 (26.9)		192 (25.8)		192 (25.8)			
Treatment protocol			0.431					0.891	
CC	303 (18.0)	172 (19.6)		148 (19.9)		143 (19.2)			
Gn	602 (35.8)	308 (35.2)		257 (34.6)		269 (36.2)			
CC plus Gn	329 (19.6)	152 (17.4)		139 (18.7)		124 (16.7)			
NC	446 (26.5)	244 (27.9)		199 (26.8)		207 (27.9)			
Year of treatment			<0.001					0.238	
2007-2009	84 (5.0)	20 (2.3)		26 (3.5)		18 (2.4)			
2010-2012	432 (25.7)	284 (32.4)		222 (29.9)		222 (29.9)			
2013-2015	519 (30.9)	311 (35.5)		260 (35.0)		260 (35.0)			
2016-2018	645 (38.4)	261 (29.8)		235 (31.6)		243 (32.7)			

BMI, body mass index; FSH, follicle stimulating hormone; LH, luteinizing hormone; AFC, antral follicle count; DFC, dominant follicle count; PTMSC, postwash total motile sperm count; CC, clomiphene; Gn, gonadotropin; NC, natural cycle.

†Ultrasonographic findings and fasting serum biochemical test performed on day 3 of menstrual cycle.

§Endometrial thickness measured with a transvaginal ultrasound scan on the day of ovulation trigger.

‡Count of dominant follicles greater than 18 mm in diameter determined by ultrasound prior to the day of intrauterine insemination.

*P values between groups were assessed by the chi-square or Fisher exact test in the unmatched cohort and by the McNemar test in the matched cohort.

### Time-to-Event Analyses

**Figure 1:**
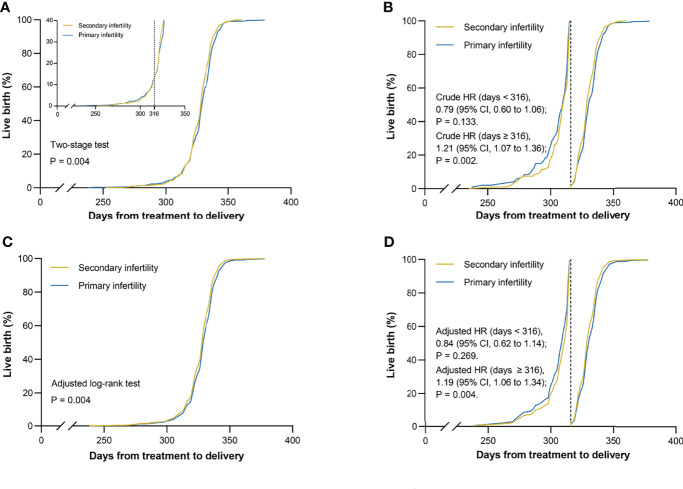
Kaplan–Meier curves for live birth. Live birth rates are shown according to infertility types in matched cohort. **(A)** Unadjusted curves crossed mainly on day 316, which was defined as the landmark point. The two-stage test was performed to compare the two groups for variability. **(B)** Unadjusted curves were cut into two parts from the landmark point. The landmark analysis was used to discriminate between events that occurred before and after 316 days of follow-up. **(C)** Adjusted curves were generated using the Cox model with the inverse probability weighting method for the following covariates: maternal age, infertility causes, treatment protocols, year of treatment, and dominant follicle count. The difference between two groups was evaluated by using the adjusted log-rank test. **(D)** Adjusted curves were divided into two parts by the landmark point and were assessed by the landmark method. HR = hazard ratio; CI = confidence interval.

During an average of 290.0 days of follow-up (431009 person-days), we identified 1143 live births in the matched cohort. Kaplan-Meier curves of live birth rates are shown in **Figure 1A**. It is clear that the crossing of the two curves occurs on day 316 from IUI to delivery. Considering the low validity of the log-rank test in this scenario, the two-stage test proposed by Qiu et al. (18) was used. Patients who diagnosed with secondary infertility demonstrated a higher cumulative live birth rate than the group of patients who diagnosed with primary infertility (*P* = .004). A similar result was obtained for the adjusted curves in **Figure 1C** using the adjusted log-rank test (*P* = .004).

### Multivariable Predictors of Live Birth

Among the 1486 matched patients (743 in each group), results of univariate analyses revealed that infertility type, maternal age, infertility causes, count of dominant follicles (DFC) greater than 18 mm to be observed prior to the day of IUI, treatment protocols, and year of treatment were selected into the final multivariable Cox proportional hazards regression model which was constructed to evaluate the relationship between eligible variates and outcome variable (**Table 2**). Patients with secondary infertility demonstrated an increase in cumulative live births (hazard ratio [HR], 1.16; 95% confidence interval [CI], 1.04 to 1.30; *P* = .007), in addition to patients who had more than one dominant follicle ≥18 mm in diameter on the trigger day (HR, 1.19; 95%CI, 1.04 to 1.35; *P* = .011). As for the causes of infertility, patients with unexplained infertility have an advantage in terms of cumulative live births compared to those classified as female factors (HR, 1.26; 95%CI, 1.10 to 1.44; *P <.*001). Besides, women who treated with NC had fewer cumulative live births compared to women treated with CC (HR, 0.80; 95%CI, 0.68 to 0.95; *P = .*013).

**Table 2:** Univariate and multivariate analysis of variable relating to live birth after propensity score matching.

Variable	Univariable			Multivariable			
	Crude HR	95% CI	*P* value	Adjusted HR	95% CI	*P* value	
Infertility types						
Primary infertility	[reference]					
Secondary infertility	1.18	1.05 to 1.31	0.004	1.16	1.04 to 1.30	0.007
Maternal age range, yrs						
<30	[reference]					
30-34	1.09	0.97 to 1.22	0.141	1.11	0.99 to 1.24	0.074
≥35	1.18	0.96 to 1.71	0.090	1.26	0.96 to 1.66	0.097
Infertility causes						
Maternal factor	[reference]					
Paternal factor	1.18	1.00 to 1.39	0.050	1.16	0.99 to 1.38	0.075
Combined	1.09	0.92 to 1.29	0.332	1.09	0.92 to 1.29	0.332
Unexplained	1.28	1.12 to 1.47	< 0.001	1.26	1.10 to 1.44	< 0.001
DFC‡						
1	[reference]					
≥2	1.25	1.11 to 1.41	< 0.001	1.19	1.04 to 1.35	0.011
Treatment protocol						
CC	[reference]					
Gn	0.81	0.69 to 0.94	0.004	0.86	0.73 to 1.01	0.070
CC plus Gn	0.81	0.67 to 0.98	0.028	0.85	0.70 to 1.02	0.076
NC	0.75	0.64 to 0.88	0.001	0.80	0.68 to 0.95	0.013
Year of treatment						
2007-2009	[reference]					
2010-2012	1.56	1.00 to 2.44	0.050	1.69	1.13 to 2.52	0.011
2013-2015	1.27	0.82 to 1.98	0.287	1.39	0.93 to 2.09	0.109
2016-2018	1.38	0.89 to 2.14	0.133	1.48	0.99 to 2.22	0.058

HR, hazard ratio; CI, confidence interval; DFC, dominant follicle count; CC, clomiphene; Gn, gonadotropin; NC, natural cycle.

‡Count of dominant follicles greater than 18 mm in diameter determined by ultrasound prior to the day of intrauterine insemination.

### Landmark Analyses

Although the unadjusted Cox proportional hazards model with infertility type as a univariate passed the Schoenfeld residual test (*P* = .388), the landmark analysis was adopted as an additional approach to validate the robustness of our results due to the presence of crossover curves in the log minus log plot is shown in **Supplementary Figure S3A**. Results of events occurring within and after the day 316 are presented in **Figure 1B**. At 316-day follow-up, the rates of live births were similar in the two matched groups (HR, 0.84; 95% CI, 0.62 to 1.14; *P* = .269). Interestingly, almost all preterm births were delivered within this time frame. However, in the follow-up from day 316 to the end point, a different finding was recorded that the rates of live births were higher in the secondary infertility group than in the primary infertility group (HR, 1.19; 95% CI, 1.06 to 1.34; *P = .*004). After adjusted for maternal age, infertility causes, treatment protocols, year of treatment, and DFC, the new model met the Schoenfeld residual test (*P* = .067) and showed two approximate parallel lines in **Supplementary Figure S3B**. To compare the results that appeared in the unadjusted model, the landmark analysis was still carried out in the new model as a sensitivity analysis and similar results were obtained, as shown in **Figure 1D**.

### Subgroup Analyses

**Figure 2:**
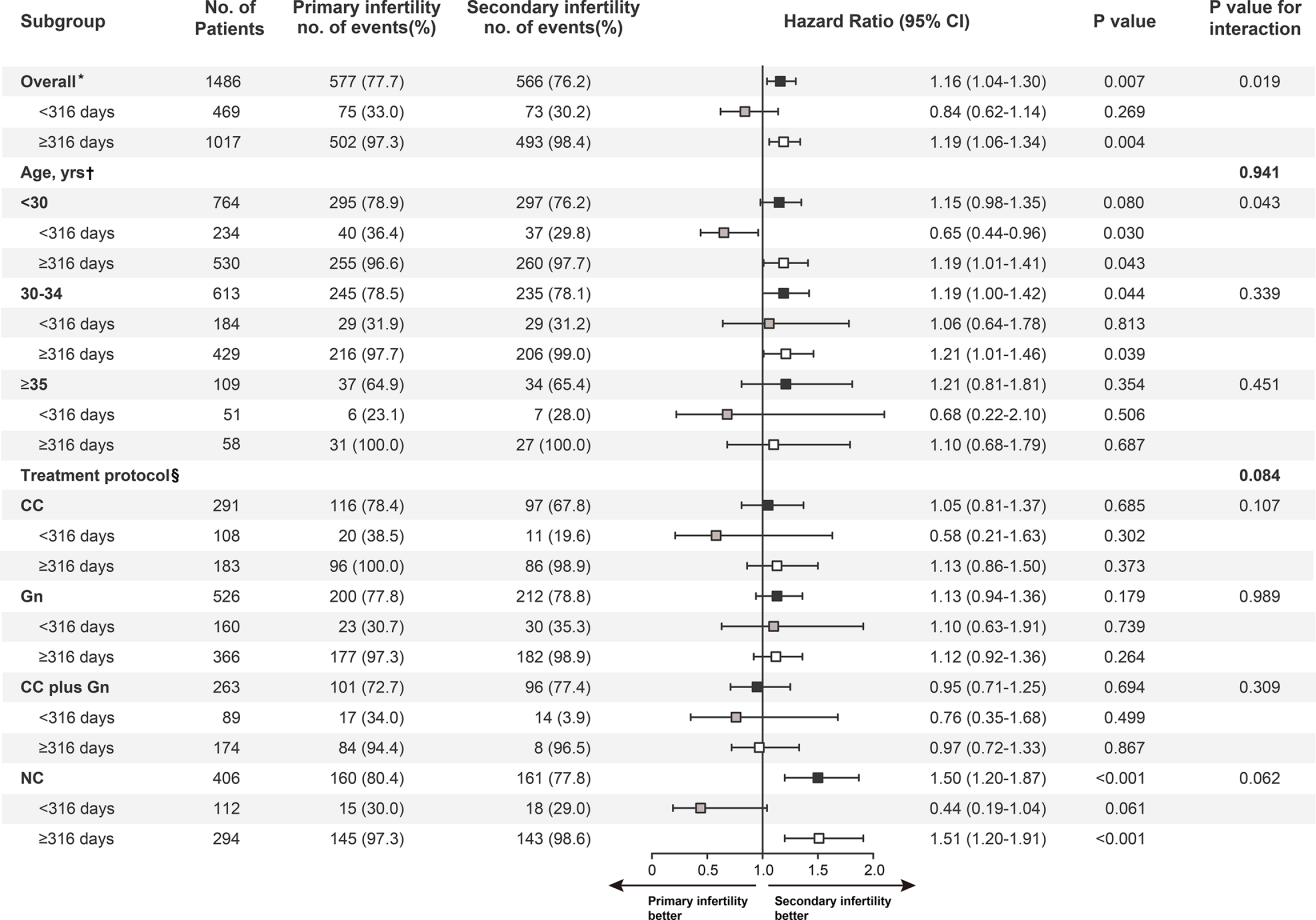
Subgroup analysis of maternal age and treatment protocol. Black boxes represent hazard-risk estimates for follow-up, grey boxes for less than 316 days after intrauterine insemination, and white boxes for 316 days to the maximum follow-up. P values for the interaction between time and infertility types with respect to the end points or the landmark point (day 316) were calculated with the use of Cox proportional hazards models. HR = hazard ratio; CI = confidence interval. *Adjusted for maternal age, infertility causes, treatment protocols, year of treatment, and dominant follicle count. †Adjusted for Infertility causes, treatment protocols, year of treatment, and dominant follicle count. §Adjusted for maternal age, infertility causes, year of treatment, and dominant follicle count.

Age is a widely known factor associated with live birth. Therefore, this trial examined the relationship between type of infertility and live birth stratified by age (**Figure 2**). For patients younger than 30 years, the live birth rate was lower in the secondary infertility group than in the primary infertility group at 316 days of follow-up (HR, 0.65; 95% CI, 0.44 to 0.96; *P* = .030), with the opposite result obtained from day 316 to maximum follow-up (HR, 1.19; 95% CI, 1.01 to 1.41; *P* = .043). In this subgroup, the live birth rate were significantly higher after the time point of 316 days after starting treatment than before; there was a significant interaction between time and type of infertility (*P* = .043). For those aged between 30 and 34 years, the secondary group showed more live births at the later period (HR, 1.21; 95% CI, 1.01 to 1.46; *P* = .039). However, no difference in live birth rates was observed between the two groups when patients were aged 35 years or older (HR, 1.21; 95% CI, 0.81 to 1.81; *P* = .354).

We also conducted a stratified analysis by treatment protocols. Effects were similar across most subgroups; however, as shown in **Figure 2**, there appeared to be more live births in the secondary infertility group among patients treated with NC and delivered 316 days after initiation of treatment (HR, 1.51; 95% CI, 1.20 to 1.91; *P* <.001).

### Sensitivity Analysis

As to unmeasured confounders in models, the results of the sensitivity analysis, whether considering full or partial follow-up, indicated that the associations between the type of infertility and live birth were robust to unmeasured confounders, except in the case of a strong unmeasured confounder that was substantially associated with live birth. As shown in **Supplementary Figure S4**, the E-values are 1.45 and 1.51 and the lower 95% CI for E-values are 1.20 and 1.25, respectively.

## Discussion

In this retrospective cohort study, the univariate and multifactorial analysis showed that more live births were observed in patients with unexplained infertility than in those with infertility due to female factors. This may be explained that almost all patients with unexplained infertility have been demonstrated to have at least one patent fallopian tube, the ability to ovulate normally, and a male partner with an adequate number of motile sperm (25–27), and therefore may have a better chance of conceiving and giving birth to live births than those with ovulatory disturbances and different pelvic and tubal disorders under the same treatment conditions.

In addition, the number of dominant follicles was a good prognostic predictor of live birth. When this number changed from 1 to 2 or more, more live births were observed. This may be due to the fact that multifollicular development may result in an increased number of fertilizable oocytes and an improved quality of the endometrium and luteal phase, leading to higher fertilisation and implantation rates (28). These findings were also confirmed in other studies (29, 30), indicating the necessity of using OS-IUI in infertile patients.

When the results of the multifactorial analysis, landmark analysis, and subgroup analysis were considered together, we found that among women who experienced their first NC-IUI cycle and were subsequently diagnosed with a clinical pregnancy, those with secondary infertility demonstrated higher live birth rates compared to those with primary infertility. For patients receiving ovarian stimulation (OS) protocols, there was no significant difference in live birth rates between the two groups. Furthermore, patients treated with OS had a greater advantage in terms of live birth rates compared to those treated with NC, which is consistent with the findings of some randomized controlled trials (31–33).Above information reminds us that patients with primary infertility may be better suited to receive the OS regimen rather than the NC regimen.

Similar effects were observed in patients younger than 35 years of age. Nevertheless, inverse associations were seen in patients who were younger than 30 years and had one or more viable infants within 316 days (i.e., preterm babies) after initiating treatment. As patients aged, the live birth rates did not differ between infertility types. That is, young women with secondary infertility who give birth at full term have a better chance of delivering a viable baby. Of note, older infertile patients (≥35 years old) seem to have a reduced probability of having a healthy baby, to the extent that no significant differences in live births were observed between the different types of infertile patients. Unfortunately, there was no strong evidence to support this conjecture in the present study. However, in Marshall’s trial (34), the conjecture has been explained by the reason that the probability of chromosomal abnormalities in the gametes of infertility patients increases with age, leading to abnormal fertilization or reduced embryonic development potential.

Over all, different results were observed for the number of live births in primary versus secondary infertile patients in different age strata after diverse treatment modalities were adopted. This may be one of the reference answers for why studies refer to infertility type and IUI outcomes were always controversial.

There are some limits in this research. First, for given its retrospective observational design and the fact that only patients who underwent their first IUI cycle and were subsequently diagnosed with a clinical pregnancy were included in this series, which may result in potential selection and confounding bias. However, it was this inclusion criterion that controlled for the impact of additional confounding factors associated with increased IUI cycles and excluded other outcome variables, allowing the study to focus only on the outcome of interest, i.e. live birth. Moreover, we used propensity score matching prior to analysis to further reduce selection and confounding bias and obtain well-balanced groups. Considering the possibility that residual confounding may still exist, we additionally conducted a formal sensitivity analysis to calculate E-values to test for the potential impact of unmeasured confounders on our results. Another weakness of this study is that we were unable to adjust our analysis as any medical condition could have been a factor in the posttreatment outcome of the discharged patients. However, all these patients were followed up regularly and strictly adhered to medical advice after IUI. Therefore, the medical condition at the time of discharge was manageable in terms of treatment outcome. In addition, due to the difficulty of controlling for the effects of various confounding factors after multiple IUIs, this article focuses only on live births in patients after initial IUI with clinical pregnancies. This may have diminished the value of this study. Finally, we did not assess newborn status in this study as we do not yet have a uniform standard regarding this aspect of recording and it may be more subjectively influenced. We plan to conduct a multicenter randomized controlled study in the future to fill these gaps and validate the results of the current study.

## Conclusion

In summary, among infertile patients who underwent a single natural or stimulated cycle followed by IUI and had a clinical pregnancy, full-term secondary infertility, mothers aged less than 35 years had a greater chance of having a viable baby than primary infertility mothers. This difference disappeared as mothers got older. In addition, similar findings were found in patients who received NC-IUI rather than OS-IUI, which indicated that primary infertility patients might have a higher live birth rate when receiving OS-IUI and therefore had a comparable presentation compared to secondary infertility ones. Our study may provide a reference for clinicians to determine rational management strategies for patients with different types of infertility in different ages.

## Data Availability Statement

The raw data supporting the conclusions of this article will be made available by the authors, without undue reservation.

## Ethics Statement

The study was approved by the Ethics Committee of the Ji’an Women and Child Health Care Hospital (Jiangxi, China). Written informed consent for participation was not required for this study in accordance with the national legislation and the institutional requirements.

## Author Contributions

WH participated in the study design and drafted the article. SC performed the analysis and wrote the manuscript. JH was responsible for the design and statistical analysis. XZ and LH made corrections to improve the final article. ZX and YQ guided writing and confirmed the final version. All authors approved the version to be published. All authors contributed to the article and approved the submitted version.

## Conflict of Interests

The authors declare that the research was conducted in the absence of any commercial or financial relationships that could be construed as a potential conflict of interest.

## Publisher’s Note

All claims expressed in this article are solely those of the authors and do not necessarily represent those of their affiliated organizations, or those of the publisher, the editors and the reviewers. Any product that may be evaluated in this article, or claim that may be made by its manufacturer, is not guaranteed or endorsed by the publisher.

## Abbreviations

IUI, Intrauterine insemination; HR, Hazard ratio; CI, Confidence interval; NCs, Natural cycles; WHO, World Health Organization; IVF, in Vitro fertilization; OS, Ovarian stimulation; STROBE, Strengthening the Reporting of Observational Studies in Epidemiology; HSG, Hysterosalpingograms; BMI, Body mass index; AFC, Antral follicle count; CC, Clomiphene citrate; Gn, Gonadotropins; hCG, human Chorionic Gonadotropin; PTMSC, Postwash total motile sperm count; SD, Standard deviation; PH, Proportional hazards; DFC, Dominant follicles.
