# Supplementary material for: Association Between Type of Infertility and Live Birth in Couples With a Single Intrauterine Insemination Resulting in Pregnancy: A Propensity Score Matching Cohort Study
Source: Front Endocrinol (Lausanne). 2022 Jul 14;13:926183. doi: 10.3389/fendo.2022.926183 (PMC9329630; doi:10.3389/fendo.2022.926183)
Supplement: Supplementary file 1 [file DataSheet_1.docx]

**Supplementary Materials**

| **Item** | **Description** | **Page** |
| --- | --- | --- |
| Table S1 | Details of R packages and their application to relevant statistical analysis in this study. | 2 |
| Figure S1 | Standardized differences before and after match. | 3 |
| Figure S2 | Mirror histograms. | 4 |
| Figure S3 | Log-log plots for the Primary infertility group and Secondary infertility group in matched cohort. | 5 |
| Figure S4 | Bias plot of unmeasured confounding on outcomes. | 6 |

**Table S1.** Details of R packages and their application to relevant statistical analysis in this study.

| **R package** | **Version** | **Relevant statistical analysis** |
| --- | --- | --- |
| “MatchIt” | 4.1.0 | Propensity score matching |
| “TSHRC” | 0.1.6 | Two-stage test |
| “cmprsk” | 2.2.1 | Fine and Gray test |
| “survival” | 3.2.7 | Landmark analysis |
| “survminer” | 0.4.9 | Adjusted log-rank test |
| “EValue” | 4.1.2 | A sensitivity analysis to access potential effect of unmeasured confounding |

**
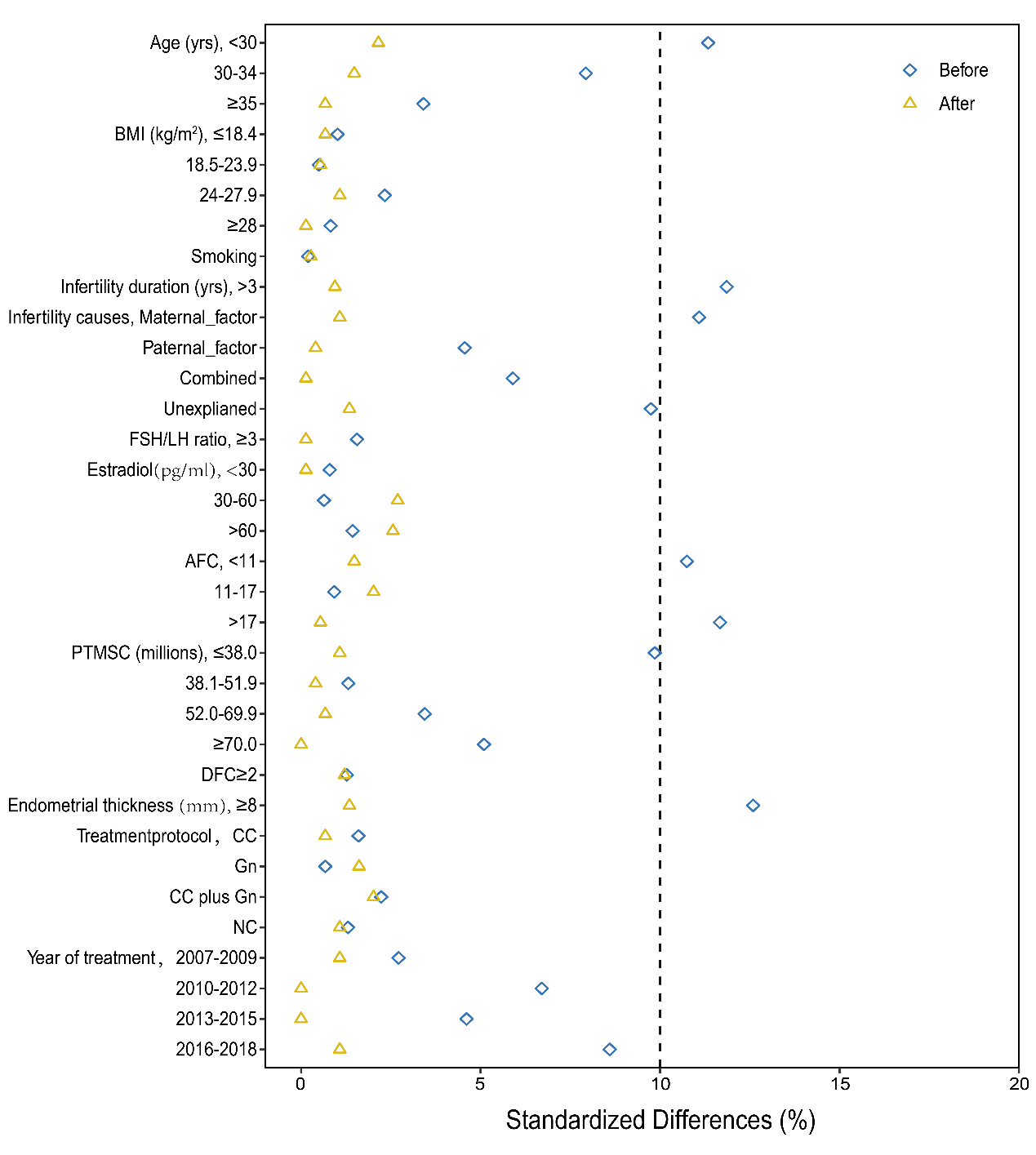
**

**Figure S1.** Standardized differences before and after matching.

**
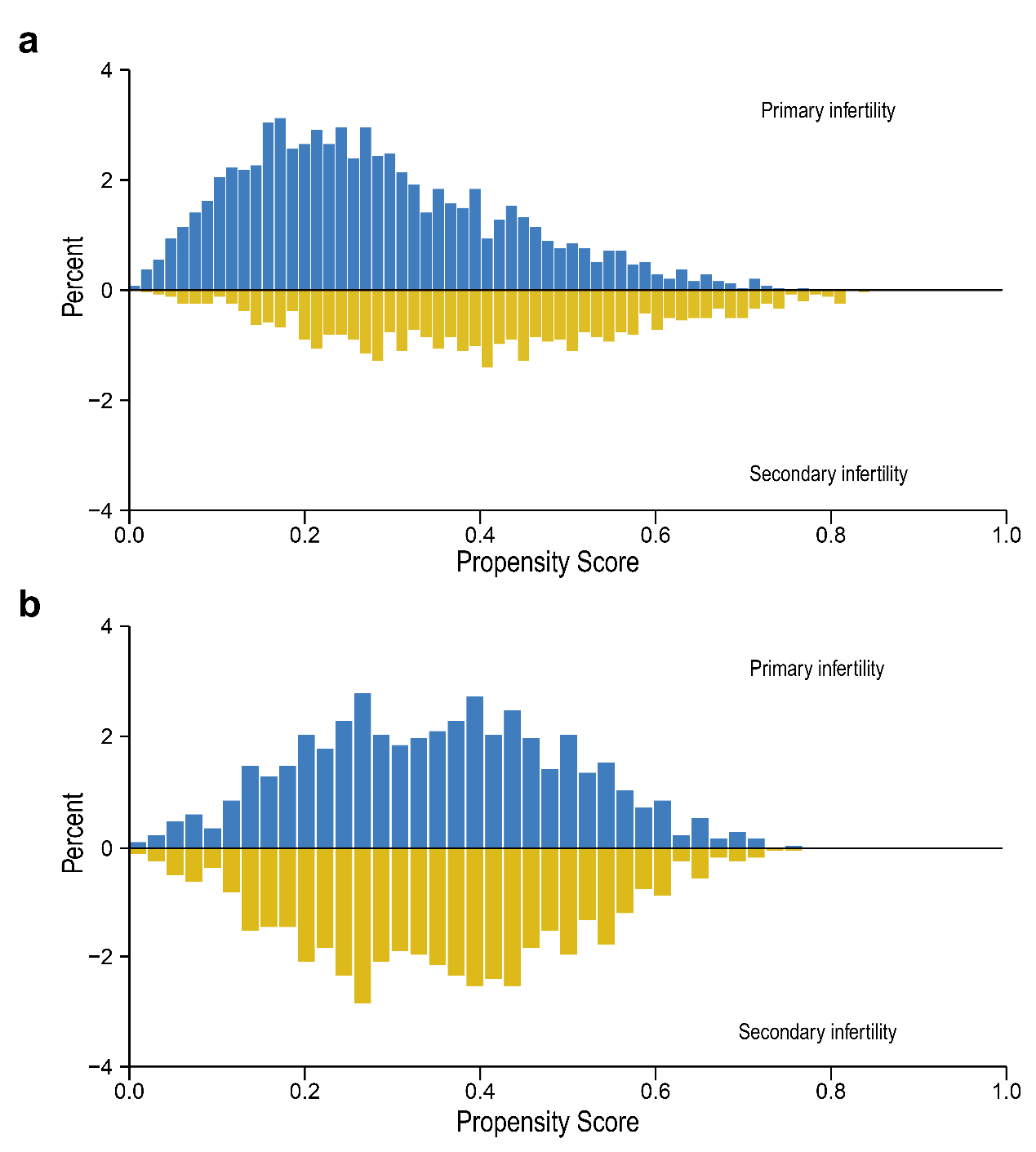
**

**Figure S2.** Mirror histograms. (a) Before matching; (b) After matching.

**
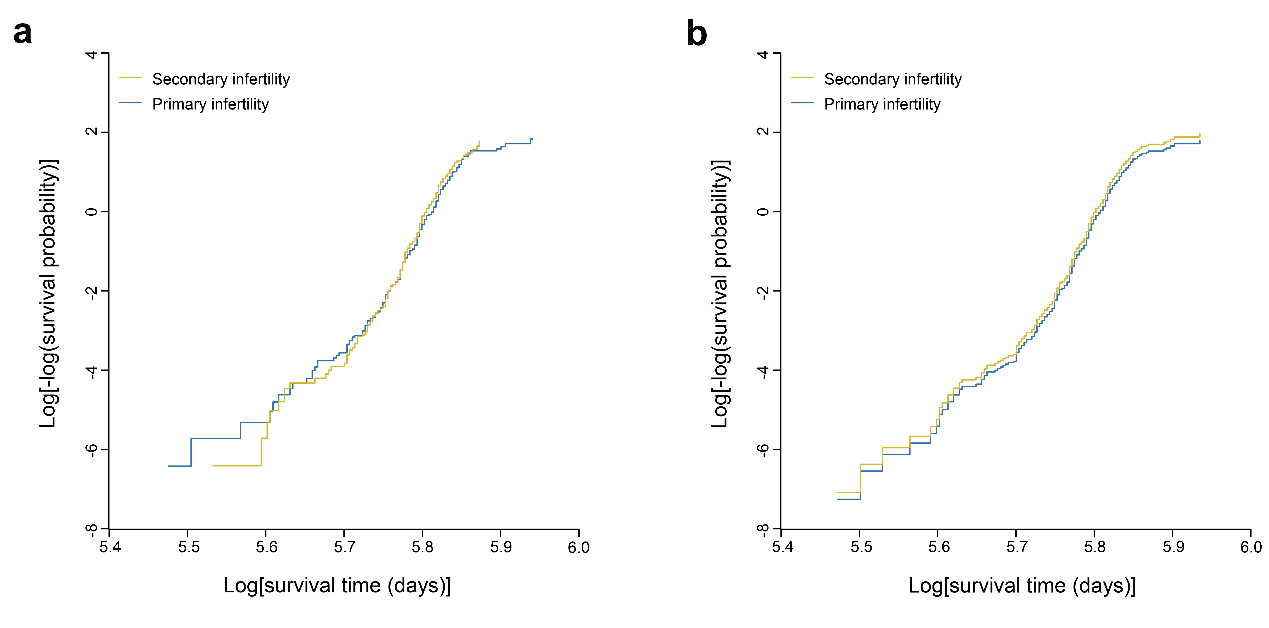
**

**Figure S3.** Log-log plots for the Primary infertility group and Secondary infertility group in the matched cohort. (a) Performed to test the proportional hazards assumption of the unadjusted model that only included the exposure variable, i.e., group. (b) Performed to test the assumption of the model adjusted for group, maternal age, infertility causes, treatment protocols, year of treatment, and dominant follicle count.

**
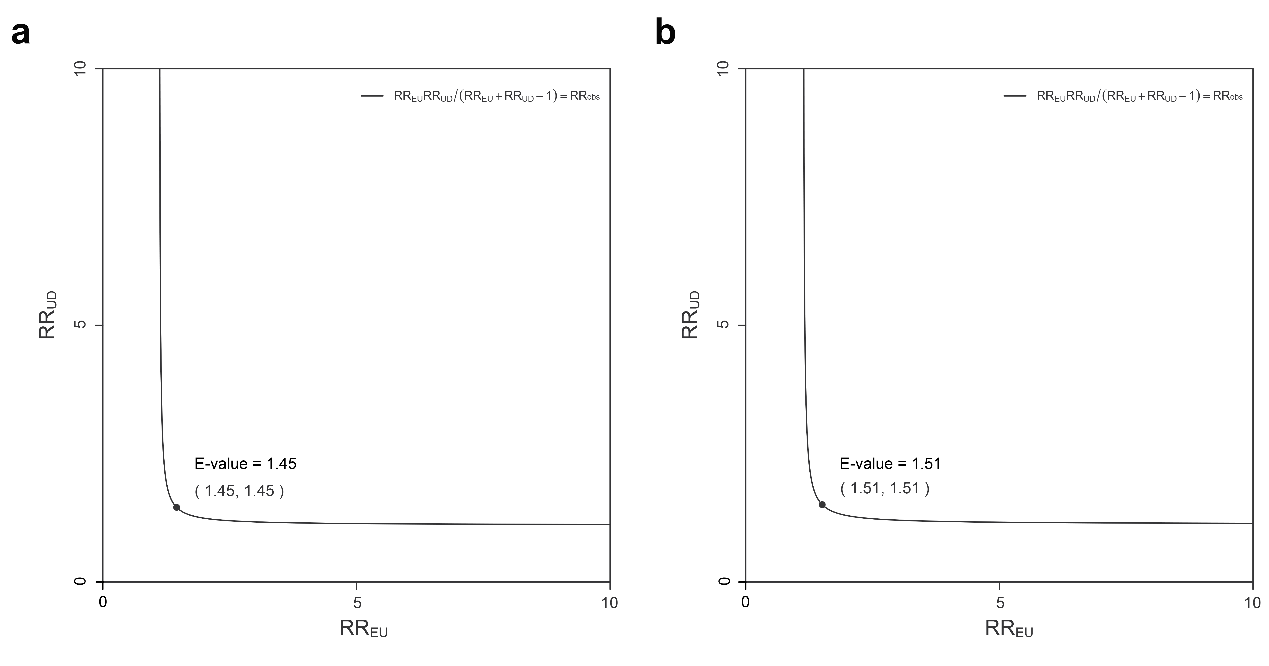
**

**Figure S4.** Bias plot of unmeasured confounding on outcomes. Figure illustrating the value of the joint minimum strength of association on the risk ratio scale that an unmeasured confounder would need to have with the infertility type and live birth to fully explain away an observed exposure-outcome risk ratio (RRobs). RREU is the risk ratio for the exposure-confounder relationship, and RRUD for the confounder-outcome relationship. The E-value mentioned in this research essentially sets the two parameters, RRUD and RREU equal to each other to see the minimum that they would both need to be. In this figure, the RRobs is also computed as (1-0.5sqrt (HRobs))/(1-0.5sqrt(1/HRobs)). HRobs is the observed HR. The HRobs for Figure a is 1.16 and for Figure b is1.19. These values can also be observed in Table 2 and Figure 2. The E-Value is estimated as RRobs + sqrt{RRobs×(RRobs-1)}. Thus, the E-value is 1.45 for Figure a and 1.51 for Figure b, which corresponds to the points (1.45,1.45) in Figure a and (1.51,1.51) in Figure b, respectively.
